# Evaluation of the Contribution of Signals Originating from Large Blood Vessels to Signals of Functionally Specific Brain Areas

**DOI:** 10.1155/2015/234345

**Published:** 2015-08-27

**Authors:** Jun-Young Chung, Yul-Wan Sung, Seiji Ogawa

**Affiliations:** ^1^Department of Biomedical Engineering, College of Health Sciences, Gachon University, 191 Hambakmoero, Yeonsu-gu, Incheon 406-799, Republic of Korea; ^2^Neuroscience Research Institute, Gachon University, 774 Namdong-daero, Namdong-gu, Incheon 405-835, Republic of Korea; ^3^Kansei Fukushi Research Institute, Tohoku Fukushi University, 6-149-1 Kunimigaoka, Aoba, Sendai 989-3201, Japan

## Abstract

The fusiform face area (FFA) is known to play a pivotal role in face processing. The FFA is located in the ventral region, at the base of the brain, through which large blood vessels run. The location of the FFA via functional MRI (fMRI) may be influenced by these large blood vessels. Responses of large blood vessels may not exactly correspond to neuronal activity in a target area, because they may be diluted and influenced by inflow effects. In this study, we investigated the effects of large blood vessels in the FFA, that is, whether the FFA includes large blood vessels and/or whether inflow signals contribute to fMRI signals of the FFA. For this purpose, we used susceptibility-weighted imaging (SWI) sequences to visualize large blood vessels and dual-echo gradient-echo echo-planar imaging (GE-EPI) to measure inflow effects. These results showed that the location and response signals of the FFA were not influenced by large blood vessels or inflow effects, although large blood vessels were located near the FFA. Therefore, the data from the FFA obtained by individual analysis were robust to large blood vessels but leaving a warning that the data obtained by group analysis may be prone to large blood vessels.

## 1. Introduction

Brain imaging via functional MRI is used to identify the functional sites that play specific roles in information processing in the brain. For an identified functional site, we expect that the site is as specific as possible to a function without exhibiting an overlap with other functional sites.

In BOLD imaging, on which most fMRI studies are based, the paramagnetic property of blood produces a bulk susceptibility difference between a blood vessel and the surrounding brain tissue, thus producing resonance frequency shifts in extravessel molecules. Thus, signals from oxygenation modulation can be expected in the tissues and for some distance into the venous side of the capillary bed [1]. This is, in itself, a short-range phenomenon located within a few tens of microns.

However, two factors make an undesirable contribution to BOLD-based fMRI signals, leading to an exaggeration of the spatial specificity and signal amplitude compared with those expected. One factor is the propagation of the bolus of oxygen-enriched blood by neuronal activation to the larger draining venous structures, which leads to the appearance of BOLD contrast in remote regions downstream of the actual site of neuronal activation [1, 2]. Another factor is the inflow effect. Neuronal activation alters vascular physiology, leading to increased flow both in the resistance pial arteries that feed the activated capillary beds and in the venules that drain these beds. MRI signals are inherently sensitive to inflowing fully magnetized spins; therefore, hemodynamic changes, particularly in larger blood vessels, can create signal fluctuations that are coincident with neuronal activation due to the inflow effect [3, 4].

Face recognition is important for social communication. The fusiform face area (FFA) is known to play a pivotal role in face processing [5, 6]. The measurement of responses of the FFA can provide crucial information regarding face recognition. The FFA is located in the ventral region [5, 6], at the base of the brain, through which large vessels, such as the inferior temporooccipital vein, middle temporal vein, vein of Labbé, middle temporobasal vein, and posterior temporobasal vein, run. The location of the FFA via fMRI varies across subjects, although the average location is almost invariant across groups of subjects. If the variation in location is dependent on large blood vessels, then the signal of the FFA can be under- or overestimated. However, this issue has not been examined. Although other functional sites also rely on individual brain structures and show variation in their location, the FFA is a special area in that it is surrounded by many large blood vessels.

In this study, we investigated the effects of large blood vessels in the FFA, that is, whether the FFA includes large blood vessels and/or whether inflow signals contribute to fMRI signals of the FFA. For this purpose, we used susceptibility-weighted imaging (SWI) sequences to visualize large blood vessels [7, 8] and dual-echo gradient-echo echo-planar imaging (GE-EPI) sequences to measure inflow signals [9, 10].

## 2. Materials and Methods

### 2.1. Subjects

Ten healthy volunteers participated in this study. After subjects were given a complete description of the study, written informed consent was obtained. This study was approved by the Institutional Review Board of Tohoku Fukushi University.

### 2.2. Measurements

All MRI experiments were performed using a Verio system (Siemens, Germany) with a standard 12-channel head matrix coil operating at 3 Tesla.

#### 2.2.1. Dual-Echo EPI

The dual-echo EPI sequence was modified from the default single-shot GE-EPI of the Siemens apparatus to acquire images at the two echo times of 13 and 38 ms, which were determined as the shortest echo times possible. For functional imaging, the dual-echo GE-EPI sequence was used with a 2000 ms repetition time (TR), 90° flip angle (FA), 220 mm field of view (FOV), 64  × 64 mm matrix size, and 3.4 mm slice thickness with 0.5 mm gaps. Twenty slices parallel to the AC-PC were acquired for each volume.

#### 2.2.2. SWI Imaging

An SWI 3D sequence was used with the parameters of TR of 28 ms, TE of 20 ms, FA of 15°, 294 × 320 matrix size, 220 mm FOV, and 1.13 mm thickness with 0.23 mm gaps. The orientation and center of slice positions were the same as those used in EPI. SWI image processing was performed using the Siemens default program.

### 2.3. Derivation of an Image for Inflow Effects

The MRI signal evolution is, after a single RF excitation, as follows [3, 11]:

(1) $$\begin{array}{c} \text{S}\left(\text{TE}\right)=\text{S}0\left(T1\right)\cdot \exp\left(-\frac{\text{TE}}{T2^{*}}\right), \end{array}$$

where S0 is the initial intensity, which is dependent on the *T*1 of a voxel and is responsible for inflow effects; TE is the effective echo time; and *T*2^*^ is the apparent transverse relaxation time. As reported in a previous study S0 is calculated directly using the MRI signals S(TE1) and S(TE2) corresponding to TE1 and TE2. The equation that yields S0 for the corresponding voxel is as follows [4]:

(2) $$\begin{array}{c} \text{S}0=\exp\left[\frac{\left\{\log\left(\text{S}\left(\text{TE}1\right)\right)-\left(\text{TE}1/\text{TE}2\right)\log\left(\text{S}\left(\text{TE}2\right)\right)\right\}}{\left(1\;-\text{ET}1/\text{TE}2\right)}\right]. \end{array}$$

### 2.4. Stimulation Procedure

Visual stimuli were images presented in the center of the visual field. An experimental run consisted of 12 event blocks of four stimulation conditions, three blocks of single stimulation for face images (SF) and scene images (SS), and three paired stimulations for face images (PF) and scene images (PS). The SF, SS, PF, and PS condition blocks were randomized at intrasubject and intersubject levels. Single stimulation blocks (SF and SS) consisted of eight different face or scene images that were presented for 50 ms, with a 1.5-s repetition period interspersed with the control state. The paired stimulation blocks (PF and PS) consisted of eight pairs of faces or scenes, with a 1.5-s repetition period between pairs and a 150 ms interstimulus interval.

### 2.5. Visual Stimulation

Visual stimuli were grayscale images presented using a projector and displayed on a mirror mounted on the head coil (spatial resolution, 1024 × 768 pixels; refresh rate, 60 Hz; Panasonic, Japan). The images occupied 3.5° × 4°, and the crosshair occupied 0.34° × 0.34°.

### 2.6. Imaging Data Analysis

The imaging data acquired from the functional session were preprocessed using BrainVoyager QX (Brain Innovation B.V., Postbus, The Netherlands) for motion correction, scan time correction, and high-pass filtering, with a cutoff frequency of 0.005 Hz. In each functional run, the prestimulus was discarded from the estimation. The 2D data from the functional session were converted into the 3D data via trilinear interpolation using BrainVoyager QX. For the multisubject analysis, 3D Gaussian spatial smoothing (full width at half-maximum, 5 mm) was applied to the data. Statistical analysis was performed using BrainVoyager QX with a procedure based on general linear modeling. Each experimental condition (with the exception of the control) was defined as a separate predictor. The default hemodynamic response function of BrainVoyager QX was the reference time course used as the predictor. This analysis was performed independently for the time course of each voxel for each subject. To complete this analysis, time series of the images obtained from each subject were converted into the native space and Z-normalized.

## 3. Results

**Figure 1:**
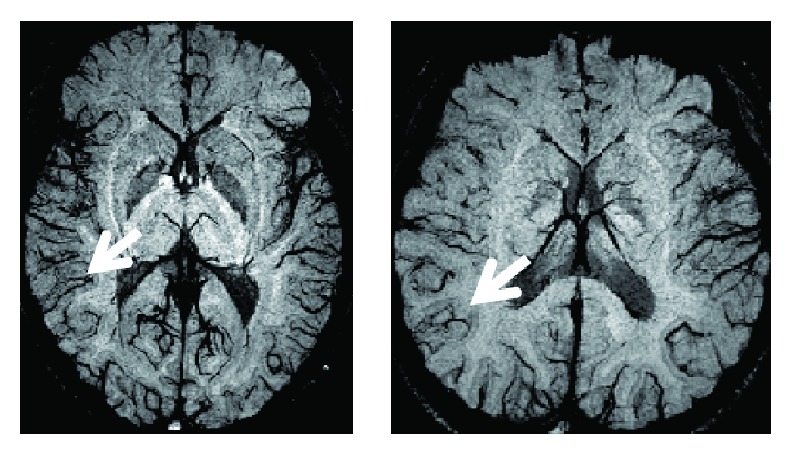
SWI-MIP images (across eight slices in sliding window mode). Image slices were from two subjects. The dark lines in the slices represent large blood vessels. The white arrows indicate some of the large blood vessels.

SWI images were acquired with minimum intensity projection (MIP) reconstruction across eight images (SWI-MIP) and without MIP. Activation maps were overlaid on SWI images without MIP (SWI-noMIP). SWI-MIP images were used to show large blood vessels more clearly. The SWI-MIP images showed blood vessels as a dark contrast, which was similar to previous reports that proposed the SWI method (Figure 1) [7, 8].

**Figure 2:**
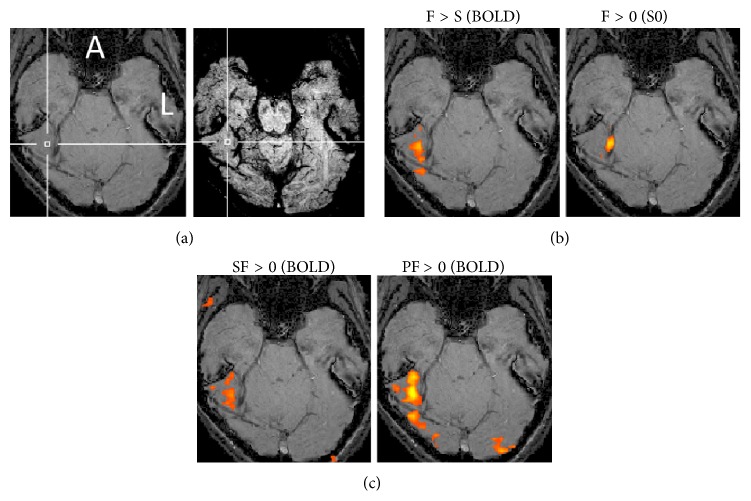
SWI images and activation maps for one subject in whom activation of BOLD and inflow signals appeared in the same image slice. The crossing of the two white lines indicates the center of the FFA. (a) SWI-noMIP (left) and SWI-MIP (right) images. (b) Activation maps on the SWI-noMIP of the FFA by BOLD signals using the contrast of the face and scene conditions (F > S) (left) and an activation site caused by inflow (S0) signals using the contrast of the face and control conditions (F > 0) (right). (c) Activation maps on the SWI image using SF > 0 (left) and PF > 0 (right). A: anterior; L: left. *P* < 0.005 (uncorrected).

**Figure 3:**
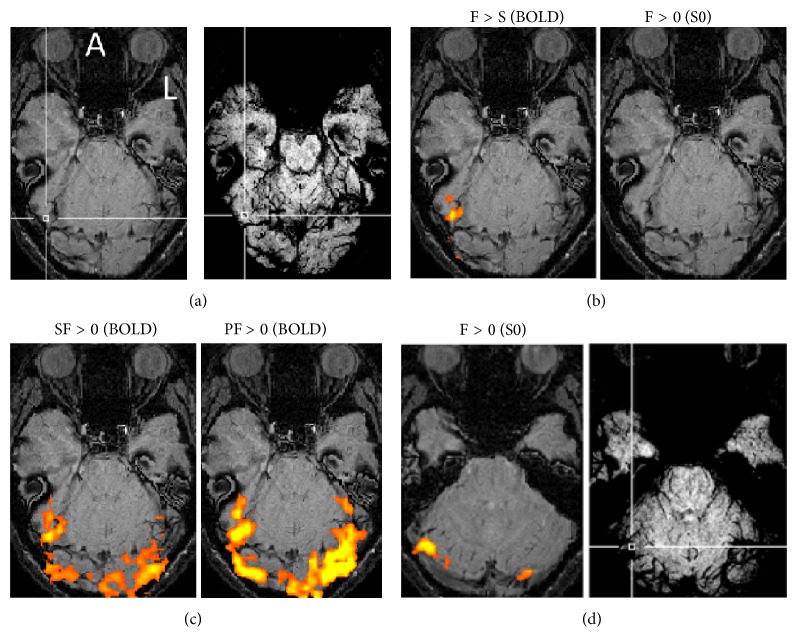
SWI images and activation maps for one subject in whom activations of BOLD and inflow signals appeared in different image slices. The crossing of the two white lines indicates the center of the FFA for (a) and the center of the S0 activation map for (d). (a) SWI-noMIP (left) and SWI-MIP (right) images. (b) Activation maps on the SWI image of the FFA were obtained by BOLD signals using the contrast of the face and scene conditions (F > S) (left), but not by inflow (S0) signals using the contrast of the face and control conditions (F > 0) (right). (c) Activation maps on the SWI image using SF > 0 (left) and PF > 0 (right). (d) Activation maps by S0 signals in a different image slice obtained by BOLD signals. A: anterior; L: left. *P* < 0.005 (uncorrected).

S0 images were acquired from images of two TEs of a dual-echo sequence. The activation map of S0 was acquired by contrasting the face conditions (SF and PF) to the control condition (this contrast included the contrast of the face versus scene conditions). Five of 10 subjects exhibited activation maps of S0 in the visual ventral region, which reflects inflow effects, while another five subjects did not. Among the data from the five subjects that showed inflow effects, variation was found in the location of activation sites (Figures 2 and 3; see Supplementary Figures  1–3 of the Supplementary Material available online at http://dx.doi.org/10.1155/2014/234345).

The FFA was identified from images with a TE of 38 ms (BOLD signals) of the dual-echo sequence by contrasting the face conditions (SF and PF) with the scene conditions (SS and PS). The data from some subjects showed that the FFA was located near the sites that reflected inflow signals. Other data showed that the FFA was located a little bit more remotely from those sites (Figures 2 and 3; Supplementary Figures  1–3). The images acquired from other subjects are shown in the supplementary figures (Supplementary Figures  4–8).

The images presented in Figure 2 are from one of ten subjects. The FFA was located at a cortical site that did not contain large blood vessels or strong susceptibility (Figure 2(a), right, and Figure 2(b), left). The activation site that reflected inflow signals was located on the right of the FFA in the same image slice (Figure 2(b), right). However, these areas did not overlap. The activation map obtained by PF was broader and stronger compared with that obtained by single stimulation (SF) (Figure 2(c), left and right).

The images presented in Figure 3 are for another subject and show the FFA and large blood vessels or susceptibility around the FFA where sites activated by inflow signals are located in different image slices. The FFA appeared to be located near large blood vessels (Figure 3(a), right, and Figure 3(b), left). Activation reflecting inflow signals was not found on the same image slice as that including the FFA (Figure 3(b), right). The activation map obtained by PF was much larger than that obtained by single SF (Figure 3(c), left and right). An activation map reflecting inflow signals was found at a lower region than the FFA (Figure 3(d), right). An activation site reflecting inflow signals appeared at large blood vessels (Figure 3(d), left and right).

**Figure 4:**
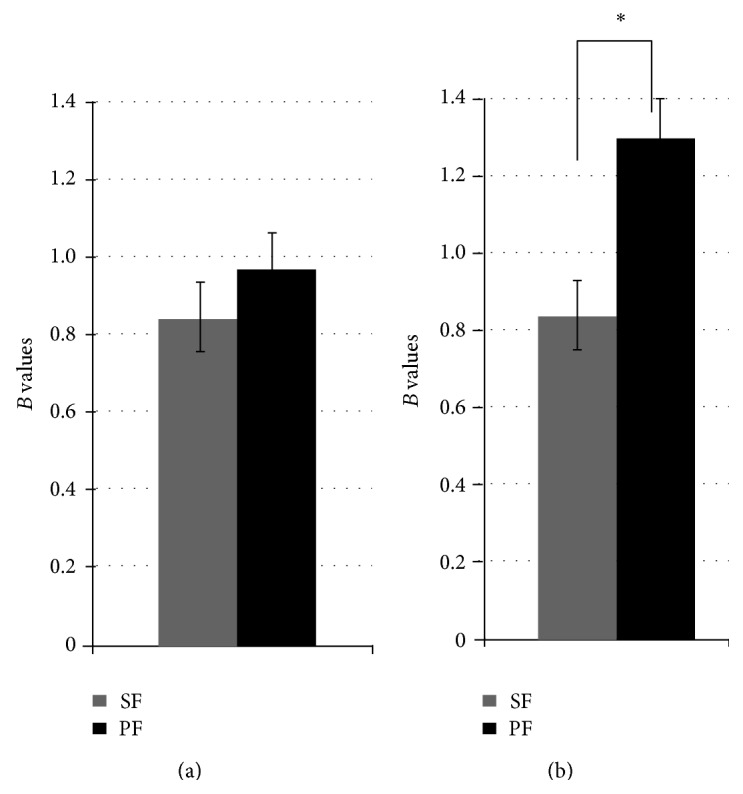
Averaged responses of the FFA in 10 subjects and estimated beta values based on BOLD signals using individual and group analyses. (a) Individual data; no significant difference was observed between SF and PF (*P* = 0.39, paired *t*-test). (b) Group data; PF was larger than SF (*P* = 0.01, paired *t*-test). Errors represent standard errors of the mean.

To assess the effect of large surrounding blood vessels in the FFA, we processed the FFA data at individual and group levels. The results of the comparison of the SF and PF conditions were different between these two analyses (Figure 4). SF and PF were not significantly different (*P* = 0.39; paired *t*-test) but PF was larger than SF (*P* = 0.01; paired  *t*-test).

## 4. Discussion

Our aim was to examine the contribution of large blood vessels to the fMRI response of the FFA. The data demonstrated, based on the presence of separate locations of activation sites of BOLD and S0, that fMRI responses of the FFA were not affected by inflow signals. The data also showed that no large blood vessels were present in the FFA. This means that the FFA was not affected directly by inflow signals and that its location was not biased by large blood vessel signals.

However, the presence of large blood vessels and the activation by inflow effects around the FFA represent a warning that the evaluation of FFA responses via intersubject averaging may lead to a wrong direction, because a representative location of the FFA acquired by averaging activated sites across subjects can pick up signals that originate from large blood vessels that surround the FFA. In fact, the comparison between an averaged signal from the locations of individual subjects and a signal from a common location based on the group-averaged data yielded different results; that is, the difference between the SF and PF conditions was larger in the group analysis than in the individual subjects' analysis (Figure 4). It is considered that broader activation maps of paired stimulation reached large blood vessels and that signals from the common area contained responses originating from large blood vessels.

The contribution of signals originating from large blood vessels to BOLD activation has been reported, and several previous studies attempted to remove this contribution or identify the location of large blood vessels [12–15]. One of the ways to achieve this is to use a spin-echo (SE) EPI sequence and compare SE-EPI with GE-EPI sequences. An SE-EPI sequence with an additional gradient can remove the contribution of large blood vessels to BOLD signal, because diffusion effects are small around large blood vessels, and the velocity in the vessels is high [13, 16]. However, the disadvantages of using these methods include small signal changes, which preclude their routine use in fMRI studies of perception and cognition, although it is helpful to determine the exact components of BOLD signals. Another reason for not using these methods is that many fMRI studies are based on low spatial resolution images.

In the present study, we used SWI sequences to acquire blood vasculature information and dual-echo EPI sequences to measure inflow effects. Imaging using SWI sequences represents anatomical scanning, whereas imaging using dual-echo sequences represents functional scanning. Therefore, there was no penalty regarding scanning time for function or regarding signal intensity, because the second echo signal can be used for typical BOLD.

Signals originating from large blood vessels may lead to misinterpretation of the functional characteristics of brain areas examined using typical fMRI, particularly in high spatial resolution studies or high functional resolution studies (which deal with subtle differences between responses to similar stimuli). Therefore, the consideration of the contribution of large blood vessels in parallel with the typical functional analysis would be needed for ensuring reliability. The present study revealed that large blood vessels do not run through the FFA and that inflow effects did not affect fMRI signals of the FFA. This assures that the fMRI data from the FFA do not include functional artifacts resulting from large blood vessels, such as the variation in the location of the FFA between subjects or the modulation of signal intensity. Conversely, information on the large vessels that surrounded the FFA provided a warning regarding group analyses. The results obtained for the FFA can also be applied to other functional areas. Therefore, it may be advisable to use SWI and dual-echo EPI sequences for high spatial resolution studies or for perception or cognitive studies that deal with subtle differences in signals [11, 17].

Our study has some limitations. Although SWI images provided a strong contrast to large blood vessels, cerebral spinal fluid (CSF) also provided a contrast of a similar level. Some additional efforts may be needed to delineate vessels if greater detail regarding the blood vasculature is required. The method used in the present study consisted of the observation of the effects of large blood vessels rather than the removal of the effects. Therefore, other methods may be used in combination with the present approach if the proposed method shows that some target areas are influenced by large blood vessels.

Taken together, the results of the present study demonstrated that the location or response signals of the FFA were not influenced by large blood vessels or inflow effects, despite large blood vessels being located near the FFA and the variation in the location of the FFA across subjects. However, a group analysis of multiple subjects may be affected by large blood vessels. This suggests that the data from the FFA obtained by individual analysis would be robust to large blood vessels, whereas the data obtained by group analysis may be prone to large blood vessels.

## 5. Conclusions

The data from the FFA obtained by individual analysis were robust to large blood vessels but leaving a warning that the data obtained by group analysis may be prone to large blood vessels. This shows that SWI sequences and dual-echo GE-EPI sequences can be used to probe functional characteristics of brain areas.

## Supplementary Material

Common description for Supplementary Figures 1-3: SWI images and activation maps for one subject in whom activations of BOLD and inflow signals appeared in different image slices. The crossing of the two white lines indicates the center of the FFA for (a) and the center of the S0 activation map for (d). (a) SWI-noMIP (left) and SWI-MIP (right) images. (b) Activation maps on the SWI image of the FFA were obtained by BOLD signals using the contrast of the face and scene conditions (F > S) (left), but not by inflow (S0) signals using the contrast of the face and control conditions (F > 0) (right). (c) Activation maps on the SWI image using SF > 0 (left) and PF > 0 (right). (d) Activation maps by S0 signals in a different image slice obtained by BOLD signals.
Common description for Supplementary Figures 4-8: SWI images and activation maps for one subject in whom activation of BOLD and inflow signals appeared in the same image slice. The crossing of the two white lines indicates the center of the FFA. (a) SWI-noMIP (left) and SWI-MIP (right) images. (b) Activation maps on the SWI-noMIP of the FFA by BOLD signals using the contrast of the face and scene conditions (F > S) (left), and an activation site caused by inflow (S0) signals using the contrast of the face and control conditions (F > 0) (right). (c) Activation maps on the SWI image using SF > 0 (left) and PF > 0 (right).
