# Supplementary material for: Evaluation of the Contribution of Signals Originating from Large Blood Vessels to Signals of Functionally Specific Brain Areas
Source: Biomed Res Int. 2015 Aug 27;2015:234345. doi: 10.1155/2015/234345 (PMC4564580; doi:10.1155/2015/234345)
Supplement: Supplementary file 1 — Common description for Supplementary Figures 1-3: SWI images and activation maps for one subject in whom activations of BOLD and inflow signals appeared in different image slices. The crossing of the two white lines indicates the center of the FFA for (a) and the center of the S0 activation map for (d). (a) SWI-noMIP (left) and SWI-MIP (right) images. (b) Activation maps on the SWI image of the FFA were obtained by BOLD signals using the contrast of the face and scene conditions (F > S) (left), but not by inflow (S0) signals using the contrast of the face and control conditions (F > 0) (right). (c) Activation maps on the SWI image using SF > 0 (left) and PF > 0 (right). (d) Activation maps by S0 signals in a different image slice obtained by BOLD signals. Common description for Supplementary Figures 4-8: SWI images and activation maps for one subject in whom activation of BOLD and inflow signals appeared in the same image slice. The crossing of the two white lines indicates the center of the FFA. (a) SWI-noMIP (left) and SWI-MIP (right) images. (b) Activation maps on the SWI-noMIP of the FFA by BOLD signals using the contrast of the face and scene conditions (F > S) (left), and an activation site caused by inflow (S0) signals using the contrast of the face and control conditions (F > 0) (right). (c) Activation maps on the SWI image using SF > 0 (left) and PF > 0 (right). [file 234345.f1.pdf]

Supplementary figures (1-8):

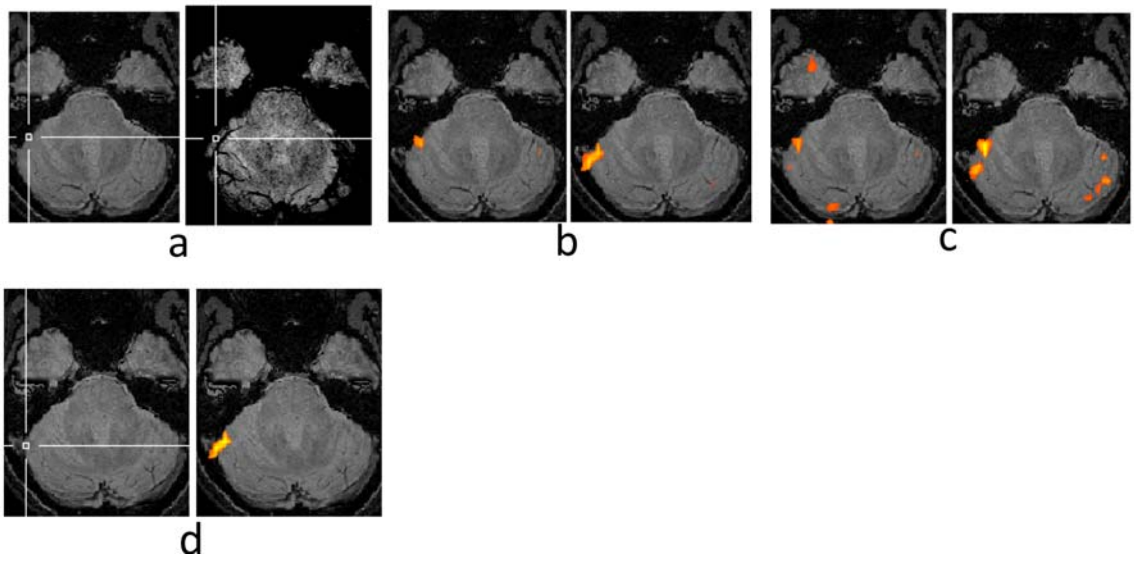

Supplementary Figure 1

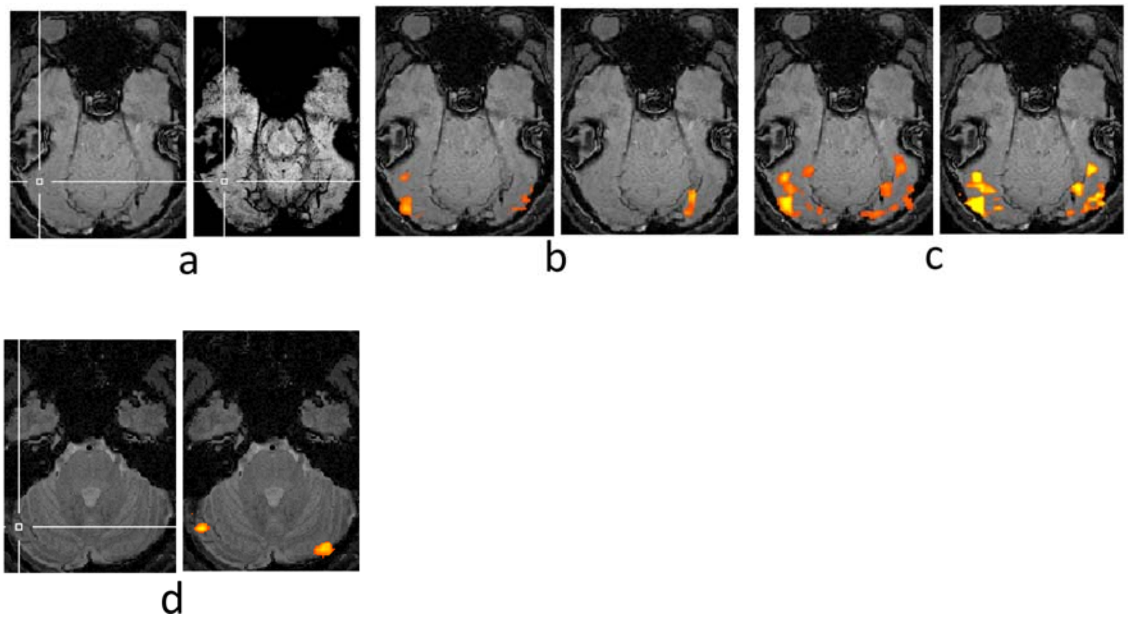

Supplementary Figure 2

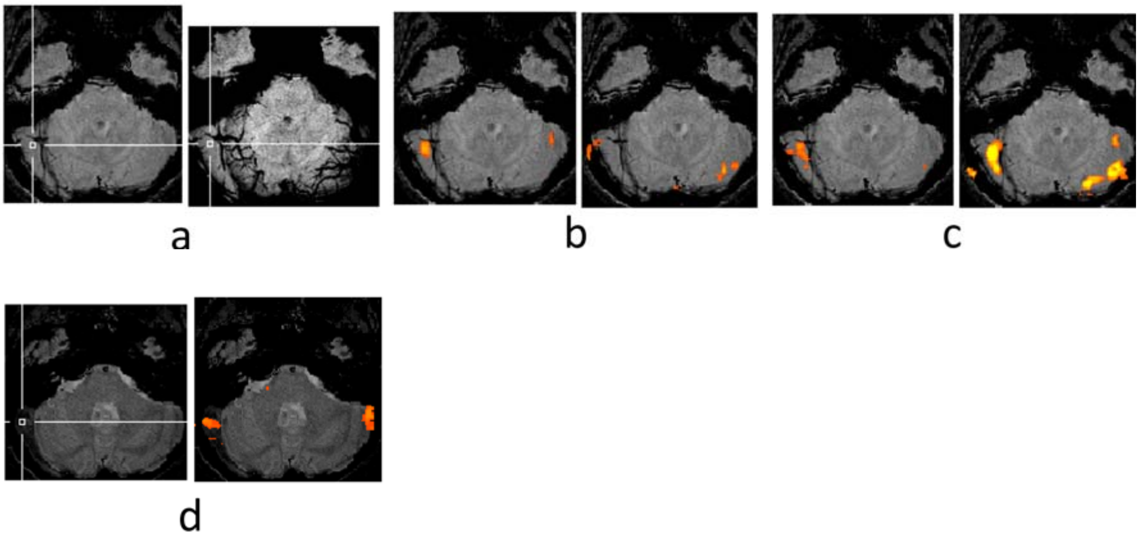

Supplementary Figure 3

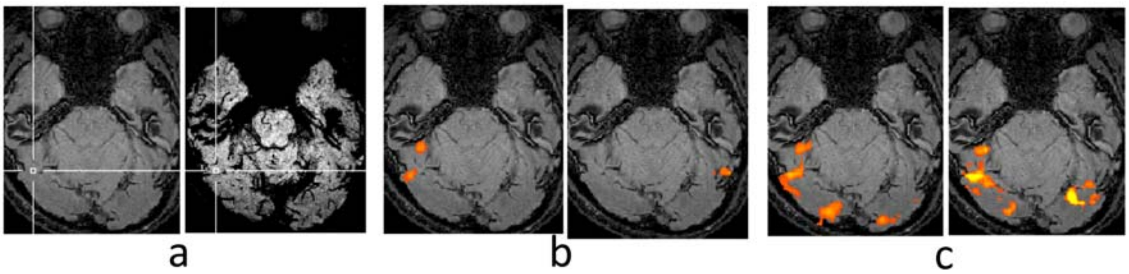

Supplementary 4

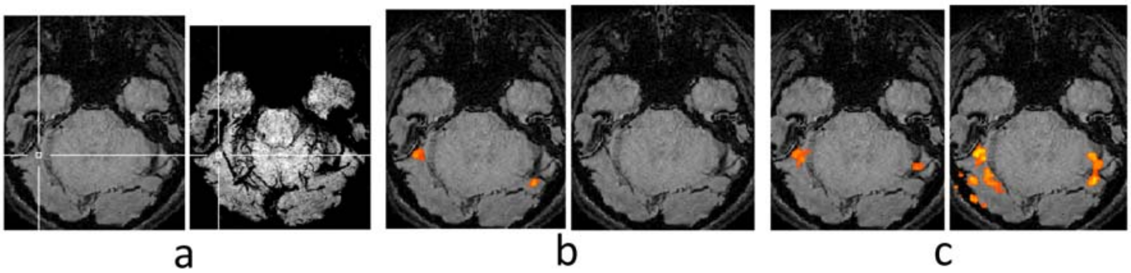

Supplementary 5

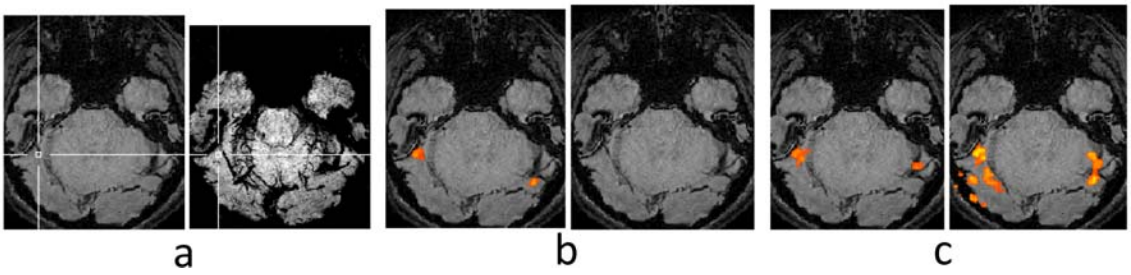

Supplementary 6

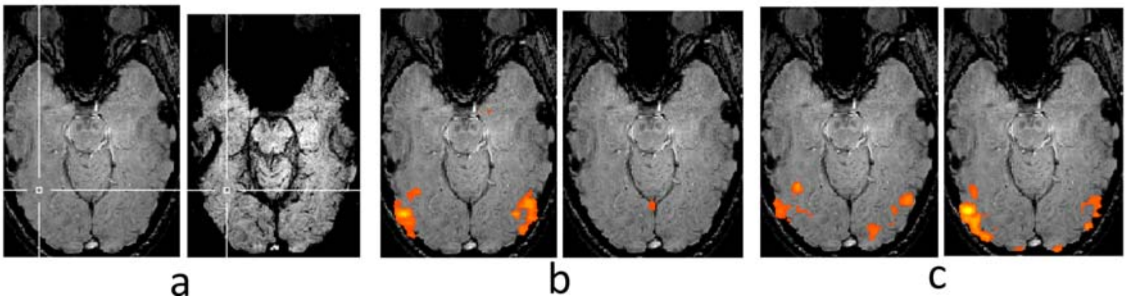

Supplementary 7

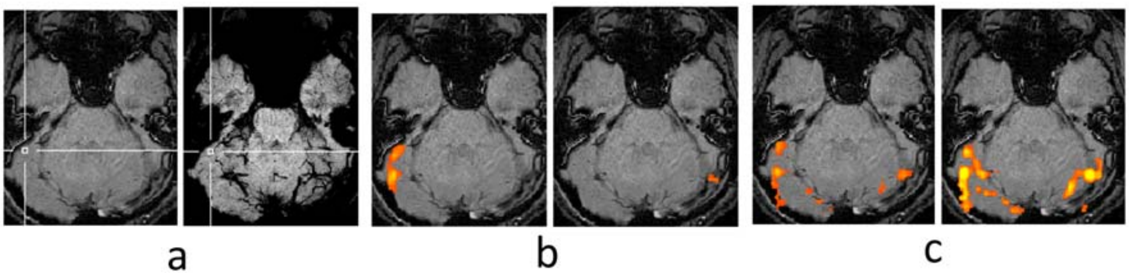

Supplementary 8
